# Correction: Shen et al. Black Phosphorus Nano-Polarizer with High Extinction Ratio in Visible and Near-Infrared Regime. *Nanomaterials* 2019, *9*, 168

**DOI:** 10.3390/nano15100703

**Published:** 2025-05-08

**Authors:** Wanfu Shen, Chunguang Hu, Shuchun Huo, Zhaoyang Sun, Guofang Fan, Jing Liu, Lidong Sun, Xiaotang Hu

**Affiliations:** 1 State Key Laboratory of Precision Measuring Technology and Instruments, Tianjin University, Weijin Road, Tianjin 300072, China; swf2014@tju.edu.cn (W.S.); connie_wind@163.com (S.H.); szytmm@tju.edu.cn (Z.S.); jingliu_1112@tju.edu.cn (J.L.); xthu@tju.edu.cn (X.H.); 2 Nanchang Institute for Microtechnology of Tianjin University, Weijin Road, Tianjin 300072, China; 3 Institute of Experimental Physics, Johannes Kepler University Linz, A-4040 Linz, Austria; lidong.sun@jku.at; 4 Key Laboratory of All Optical Network and Advanced Telecommunication Network of Ministry of Education, Institute of Lightwave Technology, Beijing Jiaotong University, Beijing 100044, China; gffan@bjtu.edu.cn

In the original publication [1], there was a mistake in Figure 5 as published. Figure 5b was accidentally duplicated with Figure 4a by the authors. The corrected Figure 5 appears below. The authors state that the scientific conclusions are unaffected. This correction was approved by the Academic Editor. The original publication has also been updated.

## Figures and Tables

**Figure 5:**
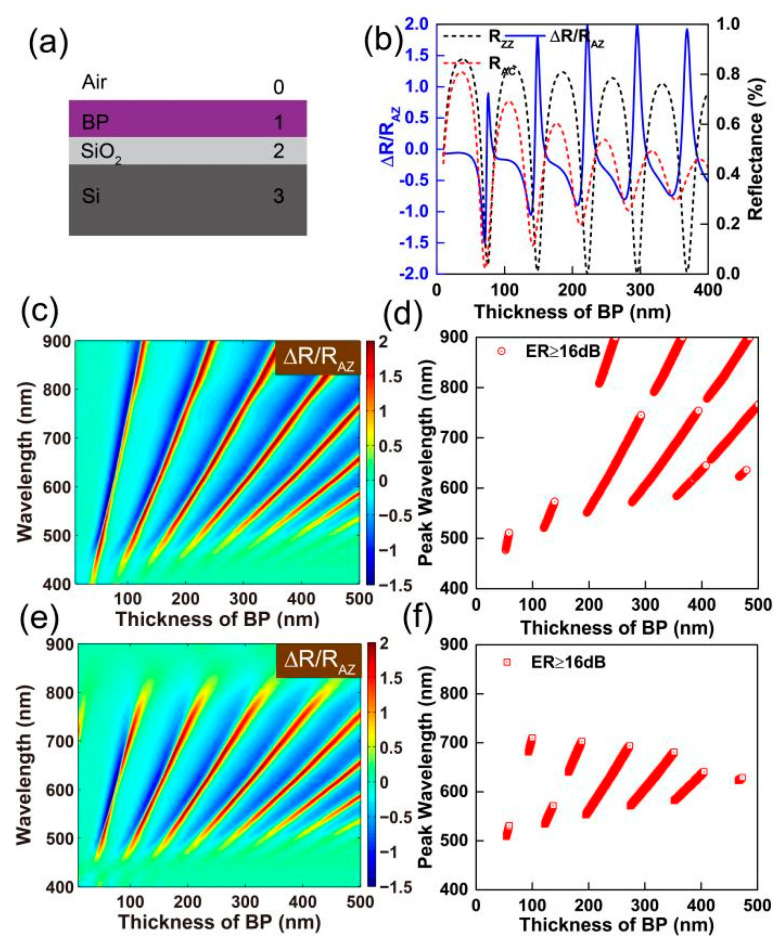
(**a**) A schematic diagram for a four-phase thin film system: air, a dielectric film (BP and SiO_2_), and a Si substrate. (**b**) The optical anisotropy of BP on a 90 nm SiO_2_/Si substrate as a function of the thickness of the BP film at a wavelength of 600 nm. Left: contour plot of ∆*R*/*R_AZ_* of BP on a 90 nm (**c**) and 300 nm (**e**) SiO_2_/Si substrate as a function of the thickness of BP and the wavelength of incident light. Right: plots of polarization wavelengths with an extinction ratio higher than 16 dB as a function of the thickness of the BP film on a 90 nm (**d**) and 300 nm (**f**) SiO_2_/Si substrate.
